# Progress of Nanocomposite Membranes for Water Treatment

**DOI:** 10.3390/membranes8020018

**Published:** 2018-04-03

**Authors:** Claudia Ursino, Roberto Castro-Muñoz, Enrico Drioli, Lassaad Gzara, Mohammad H. Albeirutty, Alberto Figoli

**Affiliations:** 1 Institute on Membrane Technology National Research Council, ITM-CNR, Via P. Bucci 17/C, 87036 Rende (CS), Italy; c.ursino@itm.cnr.it (C.U.); food.biotechnology88@gmail.com (R.C.-M.); e.drioli@itm.cnr.it (E.D.); 2 Department of Inorganic Technology, University of Chemistry and Technology Prague, Technická 5, 166 28 Prague 6, Czech Republic; 3 Center of Excellence in Desalination Technology, King Abdulaziz University, P.O. Box 80200, Jeddah 21589, Saudi Arabia; mbeirutty@kau.edu.sa; 4 Mechanical Engineering Department, King Abdulaziz University, P.O. Box 80204, Jeddah 21589, Saudi Arabia

**Keywords:** nanocomposite, mixed matrix membranes (MMMs), water treatment, nanoparticles (NPs), carbon nanotubes (CNTs) zinc oxide (ZnO), graphene oxide (GO), silver (Ag), titanium dioxide (TiO_2_)

## Abstract

The use of membrane-based technologies has been applied for water treatment applications; however, the limitations of conventional polymeric membranes have led to the addition of inorganic fillers to enhance their performance. In recent years, nanocomposite membranes have greatly attracted the attention of scientists for water treatment applications such as wastewater treatment, water purification, removal of microorganisms, chemical compounds, heavy metals, etc. The incorporation of different nanofillers, such as carbon nanotubes, zinc oxide, graphene oxide, silver and copper nanoparticles, titanium dioxide, 2D materials, and some other novel nano-scale materials into polymeric membranes have provided great advances, e.g., enhancing on hydrophilicity, suppressing the accumulation of pollutants and foulants, enhancing rejection efficiencies and improving mechanical properties and thermal stabilities. Thereby, the aim of this work is to provide up-to-date information related to those novel nanocomposite membranes and their contribution for water treatment applications.

## 1. Introduction

Polymeric membranes are widely used for water treatment, e.g., waste streams from agro-food [1], textile [2], and petroleum industries [3], or removal of pollutants from drinking water [4], enabling the concentrate to be treated or discharged and, thereby, reducing the contaminants directly or indirectly discharged into wastewater [1,5,6]. Pressure-driven membrane processes such as Microfiltration (MF), Ultrafiltration (UF), Nanofiltration (NF) and Reverse Osmosis (RO) are considered as promising alternatives for the removal of large amounts of organic micropollutants; however, NF and RO are the processes proven to be very effective filtration technologies in withdrawing micropollutants [7,8].

Over the past decade, numerous trials have been devoted to the manufacture of synthetic membranes for particular applications having appropriate features such as permeability, selectivity, and specific chemical and physical properties. To reach this target, various techniques have been performed such as track-etching, stretching, sintering, phase inversion, electrospinning and interfacial polymerization [9]. Various organic and inorganic material have been used for membrane preparation. Inorganic membranes are generally prepared using materials such as ceramics, metals and glass, while organic membranes are made from polymers or composite materials [10]. Ceramic membranes have higher thermal, mechanical and chemical stability than polymeric membranes. In addition, the hydrophilicity and the surface charge of ceramic membranes are higher than the hydrophilicity and the surface charge of the polymeric one. Hence, ceramic membranes can be used under extreme pH and temperature conditions and in high oxidizing environment [11]. On the other hand, polymers offer great design flexibility and are generally cheaper.

MF and UF use mainly polymers such as polysulfone (PSF), polyethersulfone (PES), polyacrylonitrile (PAN), polypropylene (PP), polytetrafluoroethylene (PTFE), and polyvinylidine fluoride (PVDF) as membrane materials. These materials exhibit excellent permeability, selectivity, and stability (chemical, mechanical and thermal) in water treatment applications. PSF and PES membranes seem to be among the most common materials for UF membranes. Indeed, these standard base polymers are also used in fabrication of NF and RO composite membranes, while PP and PVDF are more typical for MF membranes [12]. However, there is a need for optimizing and enhancing the separation performance of these polymeric membranes [3], as well as improving some other physical properties such as stability, hydrophilicity profile and fouling resistance [13].

Despite the breakthrough technologies in the membrane industry, some problems still need to be solved for large-scale applications. The main problem is the membrane fouling, which is the main limiting factor in industrial membrane applications [14]. The accumulation on membrane surface and inside the pores of natural organic matter and inorganic solutes forms an additional hindrance that leads to membrane fouling. Biofouling is the most intrinsically complex form of fouling. Biofouling is a consequence of irreversible microbial cell adhesion, of one or more type of bacteria, followed by a colonization of membrane surface forming microbial biofilm [15]. Once the biofilm is formed at membrane surface, it will be extremely difficult to remove using biocides [16]. Furthermore, biofilm inhibits the solvent permeation across the membrane that leads to increase the transmembrane pressure to keep the same productivity. Consequently, fouled membranes may consume a large amount of cleaning agents, which can damage the membranes surface and induce their substitution in harsh cases. Thus, these may increase the costs of operation and maintenance of the water treatment plant [17]. The suitable choice of membrane materials, pre-treatment and operating designs could attenuate, in a certain way, the fouling phenomena; however, from an industrial point of view, the durability of the membrane is still problematic and represents a challenge because of its complexity and variety [18]. For long time, the phenomena of membrane fouling has been amply studied from many points of view, such as understanding fouling mechanisms, factors and types affecting fouling growth, to mitigate their consequences. Although many efforts have been made to modify membrane surfaces for reducing the fouling, using several chemical modification techniques, e.g., by grafting hydrophilic compounds on the membrane, a satisfactory solution has not been achieved. Recent breakthrough in nanotechnologies has enlarged the range of applications to membrane technologies to improve water treatment. Polymer nanocomposite membranes are advanced membranes including dispersed nanoparticles in their polymer matrices. They could be utilized for different membrane processes such as gas–gas, liquid–liquid, and liquid–solid separation. In the 1990s, nanocomposite membranes were originally developed for the gas separation processes [19,20], where extremely selective zeolites were filled into polymers to enhance both permeability and selectivity [21]. Thanks to their properties, gas separation, sensor applications [22,23], proton exchange membrane fuel cells (PEMFCs) [24,25], direct methanol fuel cells [26], lithium ion battery [27], pervaporation (PV) [28], organic solvent nanofiltration (OSN) [29], and water treatment have been investigated using nanocomposite membranes.

Generally, nanocomposite membranes are prepared by introducing nanoparticle materials (the filler) into a macroscopic sample material (the matrix). Nanoparticles may be either coated onto membrane surface or dispersed in the polymer solution before membrane casting [30]. In this framework, secondary materials commonly known as fillers are incorporated into the main polymeric matrix to generate polymer-nanocomposite membranes which are also referred to as mixed matrix membranes (MMMs) [12] or nano-enhanced membranes [31]. The nanocomposite membranes are a potential alternative to face all these challenges. Actually, the fabrication and use of these membranes is one of the current applications of the nanotechnology in membranes for water treatment [32]. For example, nanoparticles (NPs)-based membranes have demonstrated low-fouling process through adding the inorganic particles [4]. Dispersing the NPs into the polymer generally forms these composite membranes which are also a suitable tool to improve the performance, such as permeability and selectivity, in polymeric membranes [33].

Typically, the addition of the fillers tend to change the surface properties of membranes influencing the separation performance, e.g., high permeability, stable flux, excellent rejection against foulants and better antifouling behavior [11,34,35].

There are numerous studies that have utilized different nanoparticles, such as silver (Ag) [36], titanium (TiO_2_) [37], zinc (ZnO) [38], copper oxide (CuO) ([39], carbon nanotubes (CNTs) [40], graphene oxide (GO) [41], aluminum (Al_2_O_3_) [42], silicon (SiO_2_) [43], iron (Fe_3_O_4_) [44], cobalt (Co) [45], zirconium (ZrO_2_) [46], clay nanoparticles [47] and zeolite (NaX) [48], in the development of novel nanocomposite membranes for water treatment applications. Moreover, several new types of nanocomposite membranes have been recently commercialized for a variety of filtration applications. In particular, membranes containing silver based NPs are used for RO, as reported by Sterlitech [49] and for water filtration by Lenntech [50] and LG Chem (NanoH2O composite membranes) (http://www.lgchem.com) [51,52]. The features of nanocomposite membranes to be applied for water treatment at industrial scale will be based on the production of tailored membranes with high selectivity, competitive flux, self-cleaning properties and at the same time respect of sustainability criteria in terms of environmental impacts, ease of use, flexibility and adaptability [53].

**Figure 1:**
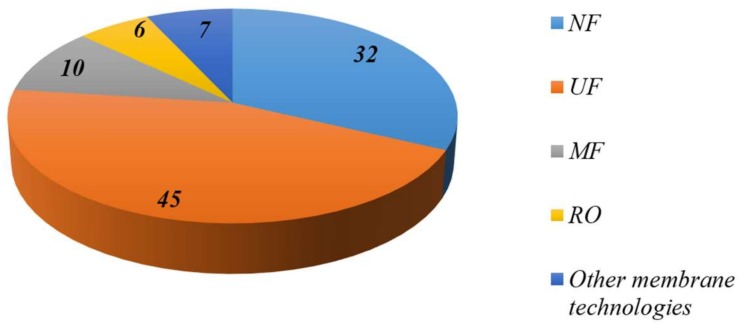
Overview about the use of nanocomposite membranes in pressure-driven membrane technologies for water treatment (data acquired from https://www.scopus.com and https://scholar.google.it/).

Figure 1 provides an outlook, based on the studies reviewed in this contribution, about main pressure-driven membrane processes where such nanocomposites membranes have been tested. The figure shows that UF technology has been the most applied technology. 

**Figure 2:**
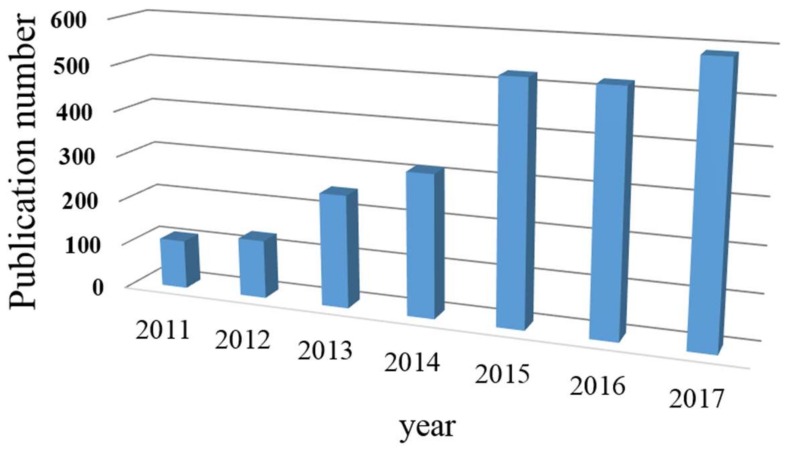
Publications related to the nanoparticles cited in this review (TiO_2_, CNTs, Ag, Cu, GO, and ZnO until 8 October 2017; data acquired from https://www.scopus.com and https://scholar.google.it/).

The use of nanocomposite is a promising alternative for water treatment that will continue to be applied based on the evidence of the growing number of publications (Figure 2) on the topic, demonstrating the increasing research interest in this field. In following sections of this review the current status of the main fillers incorporated into polymeric membranes is addressed. Table 1 displays the main uses of these membranes for water and wastewater treatment. In addition, the state-of-the art of the generated nanocomposite membranes are described, as well as an overview concerning the type of membrane technology where the membranes were applied. In this review, the advances related to the use of the most common nanofillers in polymeric membrane preparation for water treatment are described in detail. Several examples related to the preparation of nanocomposite membrane are reported and discussed in terms of rejection, antifouling and antibacterial properties, and risk to health.

## 2. Carbon Nanotubes (CNTs)

Carbon nanotubes (CNTs) have recently attracted the attention of researchers due to their extraordinary electrical, mechanical, and thermal properties and partial antibacterial activity. Indeed, they alter the physico-chemical properties of membranes, which encourage their potentiality for several applications. Typically, the inner pores of CNTs tend to act as selective nanopores; thereby, the CNT-filled membranes show an enhanced permeability without a decrease in selectivity, coupled to enhancements in mechanical and thermal properties [150]. For example, Celik et al. [40] enhanced the hydrophilicity of PES membranes blending with CNTs; the generated nanocomposite membranes (at 2 wt %) displayed higher pure water flux and slower fouling rate than the pure PES membranes. Similarly, Daraei et al. [142] enhanced the hydrophilicity of PES membranes by means of incorporating chemically modified multi-walled CNTs, showing higher pure water flux compared to pristine one; basically, the hyperbranched polycitricacid on CNTs offered many functional groups and significantly improved membrane fouling resistance against whey proteins. Thereby, the acid-modified CNTs developed surface functional groups that increased their hydrophilicity, tending to increase the rejection of hydrophobic pollutants. This was reported by Kim et al. [143] during the incorporation of such filler in polyamide (PA) thin-film composite membranes.

On the other hand, a thin-film layer enhanced the pure water permeability of the n-TFN membrane by 23%, compared to the one unfilled layer [140]. In addition, the membranes indicated greater anti-adhesive and antibacterial properties (using *Pseudomonas aeruginosa*). In the same way, coated nitrocellulose membranes with a nanocomposite containing 3 wt % of single-walled CNTs exhibited significant antimicrobial activity (80–90%) toward Gram-positive and Gram-negative bacteria as well as virus removal [141]. Moreover, these nanocomposite membranes are potential candidates for water purification because they did not present any toxicity against fibroblast cells, so they could be used as membrane filter for drinking water treatment.

The preparation of amino-functionalized-multi-walled CNTs-PSF composite membranes was carried by Shah and Murthy [144]; such membranes were tested for heavy metal removal displaying maximum removals about 94.2% and 78.2% for Cr(VI) and Cd(II), respectively, which was just 10.2% and 9.9%, respectively, with pristine PSF membranes. In the case of these composite membranes, the percentage rejection of heavy metal was found to increase with increase in loading of MWNTs. On the other hand, thin-film nanocomposite membranes embedded with poly(methyl methacrylate) (PMMA) hydrophobic modified multi-walled CNTs by interfacial polymerization were prepared by Shen et al. [145]. These membranes presented high Na_2_SO_4_ rejection (99%), and the water permeate flux was enhanced about 62% compared to the pristine thin-film composite membrane.

The introduction of polar functional groups in multi-walled-CNTs was found to be positive in order to have a good dispersion of the filler in the preparation of membranes, associated to the formation of hydrogen bonds between the solvents, the multi-walled-CNTs and the polymer [146]. This was noted in the preparation of oxidized and aminated multi-walled CNTs filled co-polymer 84 composite membranes. Additionally, the presence of the functionalized filler enhanced the permeability and reduced the fouling during the filtration of dyes [146]. Generally, most multi-walled CNTs are treated with acid to enhance their hydrophilicity before their use in preparation of composite membranes; nevertheless, the acid treatments lead to structural damages of the CNTs wall. Thus, Sianipar et al. [147] proposed the coating of polydopamine on multi-walled CNTS to enhance the hydrophilicity of the inorganic filler, and at the same time avoid its wall damage. These polydopamine-multi-walled CNTS were incorporated into PSF membranes, which maintained a high rejection performance (>99%), the water permeability was increased (by 19–50%) and they had higher mechanical strength than the pristine PSF membrane; however, they did not demonstrate a better performance than those membranes treated with acids.

The use of CNTs-filled nanocomposite membranes has not been limited to UF and NF applications only; recently, membrane bioreactors (MBR) have attracted much consideration in the field of wastewater treatment. Unfortunately, MBRs tend to have fouling issues meaning short membrane life. Thereby, Khalid et al. [148] developed PEG-CNTs composite PSF membranes for wastewater treatment using MBRs. In principle, the addition of the functionalized CNTs into PSF membranes reduced the interaction between protein and membrane surface enhancing the fouling resistance by 79%, while the performance was enhanced with four-fold increase in water and protein permeabilities. Similarly, Mulopo [149] treated bleach plant effluent in MBR using CNTs-PSF membranes. The incorporation of CNTs into nanocomposite membranes also increased the flux in the PSF membranes (i.e., up to 0.6 from 0.15 L m^−2^ h^−1^ kPa^−1^, respectively); this is attributed to the presence of the O-H bonds. Finally, these approaches showed the versatility of CNTs-filled nanocomposite membranes for water treatment applications.

## 3. Titanium Dioxide (TiO_2_)

**Figure 3:**
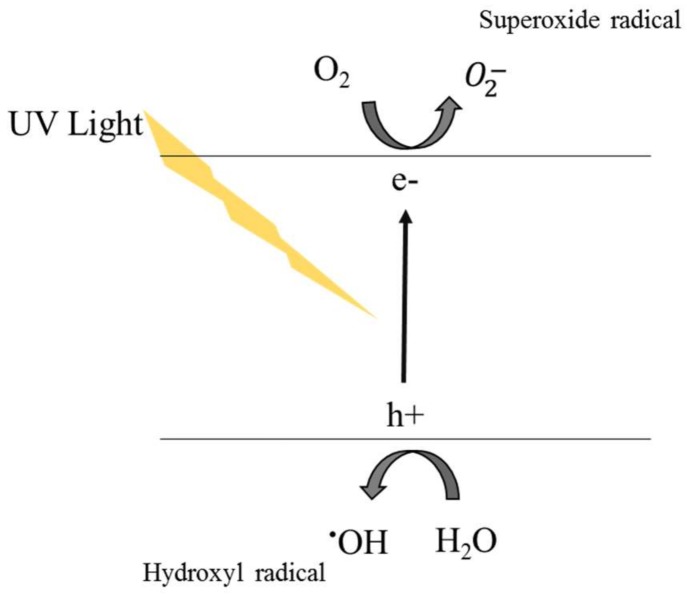
Photocatalytic mechanism of TiO_2_.

Titanium dioxide (TiO_2_) is a photocatalytic material that is currently used in a variety of applications, e.g., disinfection agent, and some other industrial uses as white pigment, food color additive, flavor enhancer and decomposition of organic compounds [32,135,151]. TiO_2_ has three different crystalline forms, namely anatase, rutile and brookite. The rutile and anatase forms, which are preferred for photocatalytic processes, can be synthesized in pure form at low temperature [152]. TiO_2_ materials are relatively less expensive than other nanomaterials, displaying good thermal and chemical stability, and low human toxicity [32]. In addition to the photocatalytic properties, they are widely studied for water disinfection and anti-biofouling. The main advantage of TiO_2_-NPs over the other ones is the nearly endless lifetime; TiO_2_ generally remains unchanged during the degradation process of organic compounds and microorganisms. As reported in Figure 3, the electron of the photocatalyst becomes excited under UV irradiation; this energy promotes the electron to the conduction band of TiO_2_, creating a pair of a negatively charged free electron and a positively charged electron hole. The electrons and holes provide strong reducing and oxidizing activities, and subsequently, they react with atmospheric water and oxygen (H_2_O and O_2_) to yield reactive oxygen species (ROS), such as hydroxyl radicals (OH), superoxide anions (O_2_^−^), and hydrogen peroxide (H_2_O_2_) [153]. The hydroxyl radicals and superoxide ions generated upon irradiation of TiO_2_ nanomaterials can react with most biomolecules, exhibiting bactericidal and virucidal activity. After a cycle of the photocatalytic reaction, the photocatalyst typically returns to its original state being ready for another excitation.

**Figure 4:**
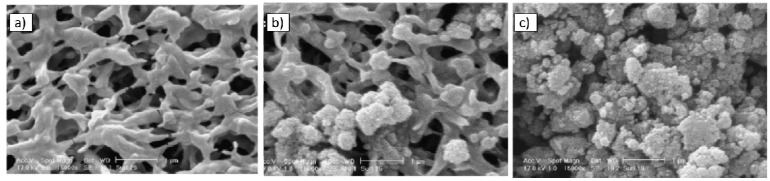
SEM images: (**a**) bare PVDF membrane; (**b**) PAA and self-assembling of TiO_2_; and (**c**) grafted by mixture of PAA and TiO_2_. Taken from [125].

Some other advantages of this nanomaterial, in terms of photo-induced hydrophilicity, and high oxidation power make it potentially attractive for applications in membrane processes. The development of self-cleaning membranes can be a way to reduce the fouling as well as maintain the membrane water permeation. Several methods are reported in the literature preparing TiO_2_-NPs filled-membranes, e.g., immobilization on membrane surface or the addition of TiO_2_ into the casting solution. Indeed, dip or spin coating, blending, hot pressing and physical or chemical cross-linking are some of the methods commonly used for incorporating NPs on membrane surface [154,155]. One of the issues during the preparation of these nanocomposites membranes is the agglomeration of the NPs. In fact, these NPs tend to form agglomerates due to their large surface area/particle size ratio. The agglomeration of particles leads to reduce the efficiency and consequentially the membrane performance. The comparison of two membrane preparation procedures using TiO_2_ was reported by Madaeni et al. [125]. The first one was conducted according to the green chemistry method, where PAA-PVDF membranes were made by grafting polymerization reaction in aqueous phase. TiO_2_-NPs (20 nm) were self-assembled on the surface of prepared PAA-PVDF by dipping the membranes in the 0.05 wt % TiO_2_ colloidal suspension. Lastly, the membranes were radiated by UV light (160 W) for binding TiO_2_ nanoparticles on the surface of hydrophobic PVDF membrane. In the case of the second procedure, 0.05 wt % TiO_2_ was added to acrylic acid monomer, then an initiator and a cross-linker reagents were also added to this solution. PVDF membranes were dipped in this reactive solution, and then the same methodology as the first method was carried out. The Scanning Electron Microscopy (SEM) images (Figure 4) show that TiO_2_-NPs are agglomerated using the first method, indicating low homogeneity and stability of NPs. On the contrary, “grafting” technique performed a covalent attachment of TiO_2_ to polyacrylic acid gel, minimizing the agglomeration of NPs, as well as strengthening the interaction between NPs and polymer network. Furthermore, the authors reported that covalent bond increased the durability of NPs on the surface of modified membranes. These results confirmed an enhanced antifouling, while the flux recovery ratio (FRR %) was 40% and 55% for self-assembled TiO_2_ on PAA grafted and PAA/TiO_2_ mixed grafted PVDF membranes, respectively.

The effect of the TiO_2_ agglomeration has been widely studied. Sotto et al. [156] analyzed the effect of NPs aggregation at low TiO_2_ concentrations. In this work, the fabrication and characterization of PES-TiO_2_ nanocomposite membrane was reported, and the effect of the addition of ethanol, as co-solvent, on the TiO_2_ dispersion was explored. TEM observation and measurement of particle size distribution revealed that the addition of ethanol promotes the increase of clusters size. Thanks to this aggregation, the membrane structure was changed from sponge-like to finger-like and the membrane permeability was improved. On the other hand, Abedini et al. [132] prepared TiO_2_/CA hybrid membranes (with an average of 62 nm) at different loading from 5 to 25 wt %; the NPs were synthesized ex situ via sonochemical method. The pure water flux experiments displayed that the flux increases when NPs concentration increase up to 20 wt % (highest flux of 47.42 L/m^2^ h). However, it was observed, at 25 wt %, that TiO_2_-NPs aggregate on the membrane top layer which lead to the decrease of water flux. 

Vatanpour et al. [157] evaluated the influence of different sizes and types of TiO_2_ (commercially available: Degussa P25, Millennium PC105 and Millennium PC500) on antifouling and performance of NF PES. The authors reported that Degussa P25 showed the best dispersion, contributing to the enhancement of the membrane hydrophilicity. This could be also attributed to the presence of 20% rutile phase, which is thermodynamically the most stable TiO_2_ crystalline form. In addition, this P25 NPs-filled nanocomposite membrane showed the highest pure water flux and superior antifouling property; in fact, the reversible fouling increased and irreversible fouling reduced by increasing of TiO_2_ content (up to 4 wt %). On the other hand, Teow et al. [158] demonstrated that anatase crystal structure displays good hydrophilicity as well. They practically compared two different types of TiO_2_-NPs, such as PC20 and P25, presenting 85 and 75 wt % of anatase structure, respectively. The antifouling properties of TiO_2_NPs-filled PVDF membranes, prepared via in situ colloidal precipitation method, were tested using humic acid (HA) as foulant. PC-20/PVDF nanocomposite membrane showed the higher permeate flux than those filled with P25, suggesting that PC-20 is more hydrophilic (the one with highest anatase content). 

**Figure 5:**
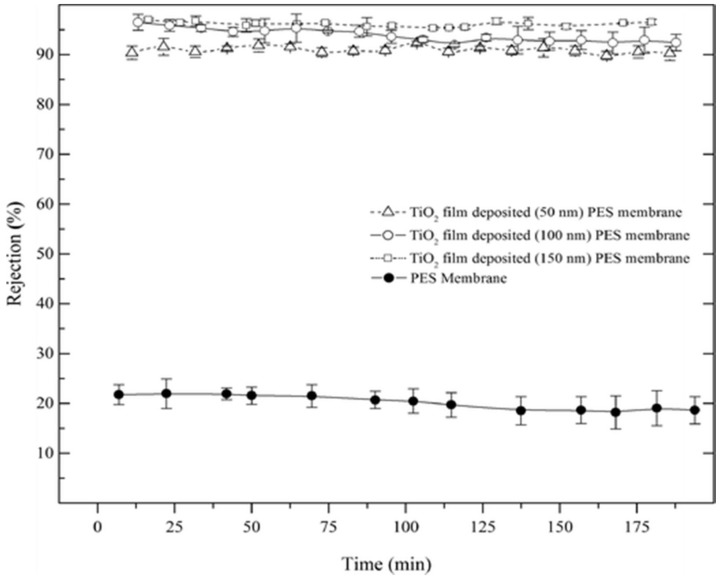
NaCl rejection of the pristine PES substrate membrane and the TiO_2_ film deposited—PES membrane. Adapted from [44].

The development of new membrane preparation procedures is also a current approach in the field. For instance, Zhang et al. [159] developed a versatile approach for the preparation of thin film composites membrane. This study compared the binding performance of TiO_2_ via the traditional self-assembly method and via self-polymerized polydopamine (PDA). Here, PDA oligomers deposited on the surface of the membrane are composed by non-covalent interactions extra- and inter-chain, including charge transfer, π-stacking, and hydrogen bonding interactions, while the -OH groups of the NPs are responsible for attaching them to the membrane surface. Another techniques has been proposed by Alam et al. [44] aiming the preparation of TiO_2_ composite membranes. The prepared TiO_2_/PES nanostructured membrane used the atomic layer deposition (ALD) technique. PES substrate membranes, prepared via wet-phase inversion method, were covered with a TiO_2_ film. The results showed that the TiO_2_/PES nanostructured membrane exhibit a NaCl rejection higher than 90%, four times greater than the PES substrate membrane, as reported in Figure 5.

Zhang et al. [160] prepared nanocomposite fibers based on PES and TiO_2_ via electrospinning technique; the potential uses of those membranes are addressed to RO. The hydrophilicity and porosity of the pristine polymer membranes were increased through the addition of TiO_2_, which agrees with the literature. Particularly, water flux was improved by 19% compared with controlled TFC membrane. Recently, the preparation of hydrothermally TiO_2_ nanofibers (inserted into PVDF membranes) under alkali conditions was successfully performed by Zhang et al. [161]. Moreover, Nor et al. [155] proposed novel flat sheet PVDF nanocomposite membrane with TiO_2_ nanofibers prepared via hot pressing method. In both studies, these membranes showed excellent antifouling properties and high photocatalytic activity for the degradation of bisphenol A under UV irradiation.

## 4. Silver (Ag)

Silver (Ag)-based compounds include Ag-NPs, stabilized Ag salts, polymer and metal oxide composites, silver dendrimer, Ag-impregnated zeolite and activated carbon materials. Generally, these materials based on Ag tend to offer antimicrobial properties that give them potential for several applications including water treatment and disinfection of medical devices. Indeed, there are some reports of silver nanoparticles incorporated into polyethylene (PE) for medical applications, and, recently, the preparation of silver nanofibers in PE, was reported by Zapata et al. [162]. In fact, silver nanoparticles are among the most often used nanoparticles for antimicrobial applications and the health and environmental consequences when silver is released from polymeric membrane must be investigated. There are some reports about the incorporation of Ag NPs into polyethylene (PE) for medical applications, e.g., Ag NPs are among the most often used NPs for antimicrobial applications. The World Health Organization’s (WHO) guidelines for drinking water reported that Ag salts can be used as bacteriostatic agents; the daily intake of Ag from drinking-water referring that Ag levels up to 0.1 mg/L could be tolerated without any risk to health [163].

**Figure 6:**
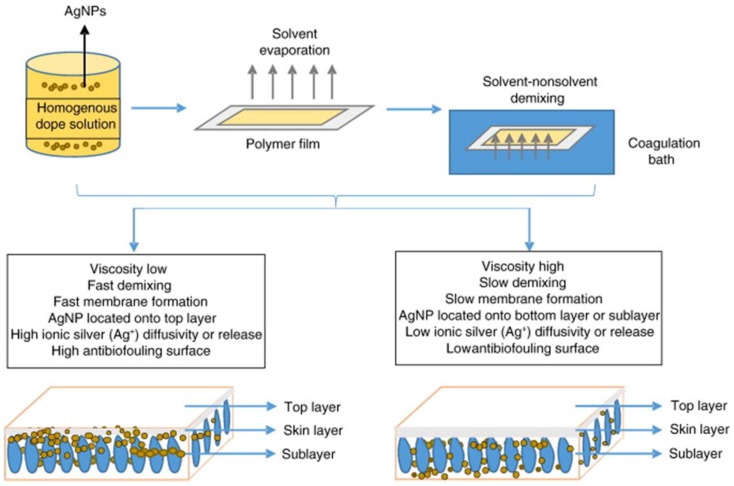
Description of the location of Ag-NP in the nanocomposite membranes developed by Sile-Yuksel et al. [104].

In general, nano-Ag, ranging from 1 to 100 nm, can be synthetized for various approaches using different precursors, reductants and capping agents [164]. The antibacterial activity of Ag-NPs has been recently discusses by López-Heras et al. [165]. Their activity generally depends on several physicochemical properties of the particles, including their size, shape, and chemistry. Typically, Ag-NPs reduce the activity of bacteria due to a synergistic effect between direct particle-specific biological effects and the release of Ag+ ions. Furthermore, Ag-NPs can stick to the bacterial cell which influences negatively the permeability and respiration of the bacteria, but particles affect the cell membrane resulting in cell lysis. In this way, the Ag particles can go through the bacterial cytoplasm, causing damage to the DNA [4,166,167]. The preparation of nanocomposites membranes using Ag has been reported by several authors [104,163,165], being cellulose acetate (CA), chitosan, polyacrylonitrile (PAN) and polysulfone (PSF) the most popular polymeric materials used for the preparation of Ag nanocomposite membranes. Sile-Yuksel et al. [104] studied the effect Ag-NPs location in polymer types. The authors presented a detailed description (Figure 6) of how the location of Ag-NP changes depending on polymer type, and, subsequently, this location influences the antibacterial properties of the nanocomposite membranes. Three different polymers, PES, PSF and CA, were used to fabricate nanocomposite membranes at three Ag-NPs different ratios (0.03, 0.06 and 0.09 *w*/*w*). The authors reported that Ag-NPs are homogeneously located along the membrane matrix both skin layer and sublayer but they protruded from the top surfaces of PSF and PES membranes. On the other hand, the increase of Ag-NP/polymer ratio tended to increase water permeability in PSF (200 > 215 > 225 > 235 L/m^2^ h bar) while it decreased using PES and CA polymers, e.g., 360 < 325 < 250 < 220 and 80 < 67 < 46 < 40 L/m^2^ h bar for PES and CA, respectively. 

The Ag-NPs incorporation on the membrane surface can be performed by direct absorption reduction, where Ag-NPs are incorporated during casting solution preparation, or in situ synthesis. Basically, ionic silver is reduced during phase inversion processes [168]. The incorporation of Ag-NPs onto the surface of sulfonated PES membranes using vitamin C as reducing agent was reported by Cao et al. [169]. Zhu et al. reported a procedure for immobilized ionic Ag^+^ and metallic Ag^0^ silver on chitosan (CS) membranes to visualize their anti-biofouling performance [170]. In addition, the antibacterial effect was evaluated using *E. coli* and *Pseudomonas*, which are generally responsible for promoting biofouling by secreting an extracellular polysaccharide. While CS membrane could not inhibit the growth of both bacteria, both the CSAg^+^ and CSAg^0^ membranes showed significant antibacterial performance. The anti-biofouling properties were studied for 10 days (using high concentration of bacteria suspensions ~10^9^ CFU/mL). Furthermore, the CS-based membrane with Ag^0^ seemed to be more stable than the one CSAg^+^ membrane.

**Figure 7:**
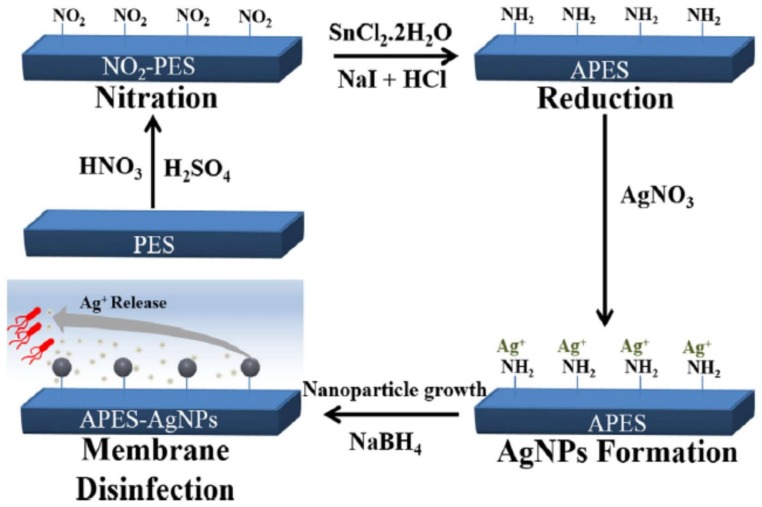
Schematic illustration of aminated-polyethersulfone (APES) membranes decorated with AgNPs, adapted from Aider et al. [171].

Recently, Haider et al. [171] reported the immobilization of Ag-NPs on PES membranes by introducing amino groups, which contribute to form aminated PES (NH_2_-PES, APES). Figure 7 shows an overview of the immobilization mechanism. The antibacterial activity was evaluated against *E. coli*. The Ag-NPs attachment on the surface of aminated PES enhanced of bacterial/silver contact, and Ag-NPs-APES show practically the highest disinfection potential by reducing the colony count to zero.

Similarly, the surface modification by using Ag-NPs of PES membranes was reported by Biswas and Bandyopadhyaya [172]. In this work, PES membranes were sulfonated using concentrated sulfuric acid to generate -SO_3_H groups on membrane surface which dissociates to give SO^3−^ and H^+^ ions. The addition of AgNO_3_ led to replace H^+^ ions by Ag^+^ ions, and the Ag-NPs-PES membranes displayed an almost constant permeate flow rate (3.45 L/h) due to a complete *E. coli* cell-killing. The influence of the preparation of AgNPs membranes, via in situ or ex situ synthesis, has been deeply discussed [100,173,174]. Regardless of the method of preparation, both methods have exhibited good antibacterial activity against *E. coli* and *S. aureus*. The measure of Ag leaching from the membrane surface indicated that the ex situ NF membranes had lower Ag release than the in situ membranes [173]. The coated Ag-NPs membranes (ex situ) had no significant influence on the permeability, pore size and cross-section morphology of the membranes in comparison with the membranes prepared by the solution blending method (in situ). The latest generally increase the solution viscosity leading the increase of the thickness of the membrane itself [100]. In addition, the NPs can be formed in the polymer solution, but have limited growth due to the solution viscosity. Andrade et al. [174] observed that, when Ag-NP synthesis occurred in situ, the Ag-NPs presented nanocubic and spherical morphologies (38 nm average edge length) that were preferentially distributed on the top and bottom surfaces of the membrane [174]. It is important to mention that addition of AgNO_3_ increased the size of NPs, while decreasing NP size enhances the antibacterial activity.

**Figure 8:**
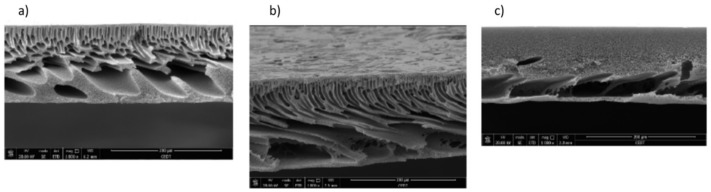
Cross-section images of: (**a**) PES membrane; (**b**) PES-AgNPs (0.32 wt %) membrane; and (**c**) PES-AgNPs (0.64 wt %) membrane, adapted from Rehan et al. [103].

An ex situ method was reported by Rehan et al. [103]. The synthesis of Ag-NPs was carried out by using chemical reduction of Ag nitrate with fructose and using PVP as capping agent. All membranes were prepared via NIPs process using PES as polymer. The amount of the Ag-NPs changed was setting from 0 to 0.64 wt %; in this way, Ag-NPs were dispersed homogeneously in the polymer matrix, the addition of particles tended to suppress of macrovoids in comparison with native membrane. This can be attributed to the increase of viscosity of the dope solution and subsequently the decreasing of the solvent/non-solvent exchange, as reported in Figure 8.

The presence of Ag-NPs on the membrane surface resulted in enhancing the antibacterial effect. The authors reported that there could be a probable interaction between NPs and the -SO_2_ functional group of PES; the interaction can also prevent the release of Ag-NPs in permeate. Moreover, the pristine and nanocomposite membranes were tested with urban wastewater (rich in bacteria) and seawater (poor in bacteria) filtration; the incorporation of Ag-NPs in PES matrix reduced the flux (normalized flux J/J0) decline from 71% to 64% for sea water, and from 74% to 60.32% for urban wastewater. Antibacterial and anti-biofouling activity were evaluated against *P. aeruginosa* and *E. coli.* The inhibition zone enlarged by increasing Ag-NPs concentration, and Ag-NPs-PES membranes inhibited the bio-film formation [103].

**Figure 9:**
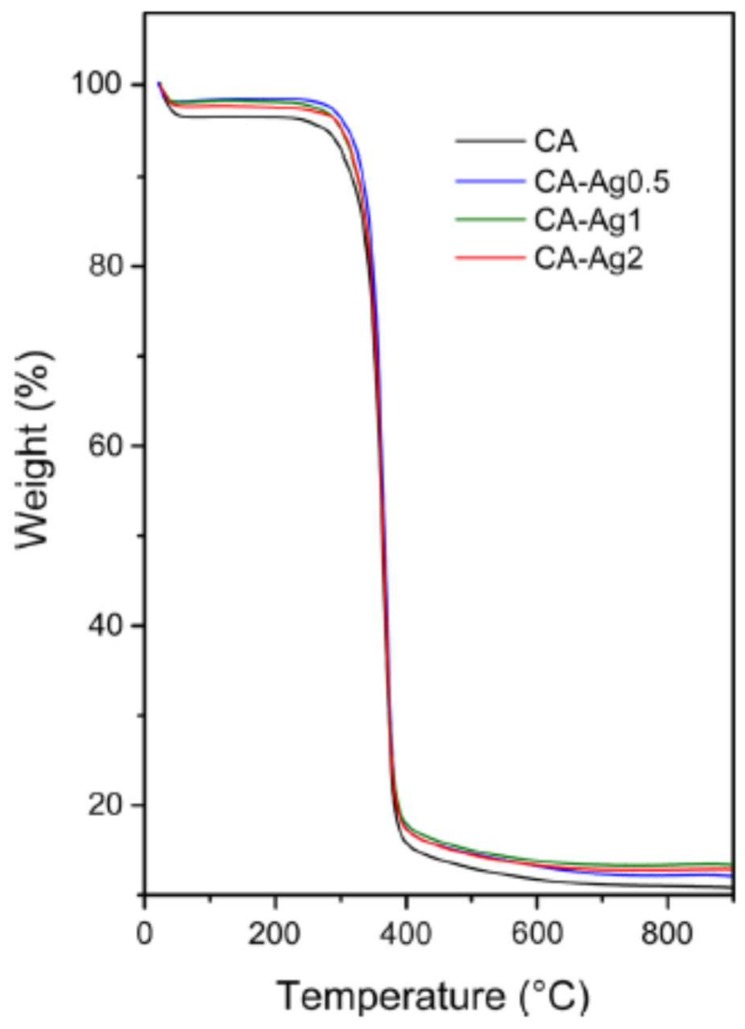
Thermogravimetric curves for pure CA and AgNP-β-CD membranes, taken from Andrade et al. [107].

Preparation of nanocomposite Ag/CA membranes via wet-phase inversion process using stabilized silver NPs was recently reported by Escobar et al. [64] and Andrade et al. [107]. Both studies demonstrated the increase of the hydraulic permeability in the membranes through the incorporation of Ag-NPs, e.g., an increase in the hydraulic permeability from 39.6 (in pristine CA membrane) to 61 kg/m^2^ h bar (at 0.4 wt % nanocomposite) [64]. Andrade et al. [107] prepared CA membranes containing different amounts (0.5–1–2 wt %) of β-cyclodextrin stabilized Ag-NPs (Ag-NP-β-CD). The pre-synthesized NPs (28 nm mean diameter) were dispersed in a dope solution to promote the effective anchoring of the NPs in the CA matrix. The thermal stability of the polymers was determined by thermogravimetric analysis (TGA), showing that the addition of Ag-NP-β-CD in the CA matrix did not compromise the thermal stability of the membranes, as reported in Figure 9. To evaluate the membrane susceptibility to bacterial adhesion, pristine and Ag-NP-β-CD membranes were exposed to *E. coli* for 48 h. The antibacterial activity was greater than 87% in each case.

Nanocomposite PVDF membranes through in situ formation of Ag-NPs was reported by Tang and Cao [175]. In this work, the antibacterial activity of the composite films with different amounts of silver nitrate (1, 5 and 10 wt %) was confirmed. The Ag ions could diffuse from the film to the nutrient agar reducing the growth of the bacteria. Thin film composites membranes were recently prepared for RO technology by Ben-Sasson et al. [109] and Yang et al. [110]. In the case of Yang’s study, a green and facile method to immobilize silver ions by the reducing catechol groups in a polydopamine (PDA) coating layer, without the use of reductants and chemicals, was suggested. The authors reported that the electrons released by the oxidation of catechol to quinone can reduce silver ions in the solution phase, and, at the same time, the O- and N-based ligand sites in PDA could serve as anchors for the resulting AgNPs. Furthermore, high flux (~65 L/m^2^ h) and comparable NaCl rejection (~85%) were obtained by using PDA coating; the nanocomposite membranes showed also clear antimicrobial effects on model bacteria *Bacillus subtilis* and *Escherichia coli*. An interesting study for preparing nanocomposite Ag-NPs-PSF membranes with enhanced permeate flux and antifouling properties was reported by Alpatova et al. [102]. 

**Figure 10:**
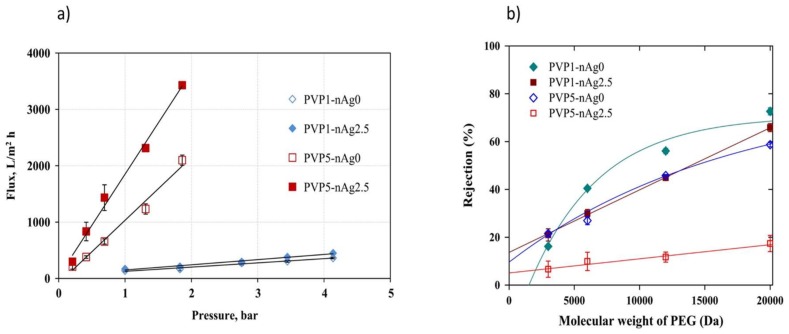
(**a**) Pure water flux of the membranes with and without 2.5% AgNPs and with 1% and 5% of PVP concentration; and (**b**) PEG rejection of membranes with and without 2.5% Ag-NPs and with 1% and 5% of PVP concentration, adapted from Alpatova et al. [102].

The effect of PVP (1–5 wt %) and Ag-NPs (0–0.05–0.1–2.5–5–10 wt %) concentrations on the morphology, performance, and antifouling/anti-biofouling properties of the nanocomposite membranes was investigated. The antibacterial tests against *P. aeruginosa* showed that membranes loaded with 2.5 wt % of Ag-NPs displayed antibacterial activities. The authors also reported that permeate flux increased at high PVP concentration from 1 to 5 wt %, thanks to the formation of more macrovoids and more porous membrane structures [103]. As Figure 10 shows, the addition of Ag-NPs improved the permeate flux while the rejection of PEGs increased at higher molecular weight PEG for all tested membranes. The addition of Ag-NPs affected the selective property through its decrease in the rejection properties, which can be attributed to the increase of pore sizes (around 5.4 µm).

Typically, studies tended to use commercially available Ag-NPs, however, there are several works about the use of Biogenic silver NPs (bio-Ag^0^) which were synthesized by *Lactobacillus fermentum* LMG 8900 [101,115,116,117]. The authors reported that Bio-Ag^0^ displays very high stability in aqueous solution and the attachment of bacterium fragment on the surface of NPs contribute to prevent the Ag-NPs aggregation. Moreover, the incorporation of bio-Ag^0^ increased the permeate flux and the hydrophilicity as well. Recently, the use of silver based-metal-organic framework (MOF) has been studied aiming to mitigate the biofouling in thin film nanocomposites for forward osmosis (FO) TFC membranes [176]. Ag-MOF nanocrystals provided a biocidal activity during six months, showing an improvement in biofouling resistance, with lower decrease in flux (about 8% in comparison with 21% of the control membranes, 24 h testing).

## 5. Copper (Cu)

Copper (Cu) and copper compounds have been demonstrated to possess bactericidal and fungicides effects against different types of microorganisms, viruses and algae [177,178]. The action mechanism of Cu ions is not yet clear, but there are some hypothesis, e.g., Cu-NPs can interact with the bacteria by generating reactive oxygen species (ROS), lipid peroxidation, protein oxidation, DNA degradation, and generation of superoxide anions [179]. Furthermore, Cu^2+^ ions may interact with phosphorus or -SH groups contained in biomolecules such as DNA and proteins; they can act by disrupting biochemical processes, leading the protein denaturation [180,181]. The antibacterial property of Cu, low cost and availability with respect to silver [124] suggest its application in several areas such as manufacturing of medical devices, textile industry [182], food packaging and water decontamination, e.g., the production of copper coated feed spacers for biofouling control [183,184].

Several techniques have been used to prepare antimicrobial nanocomposite membranes using Cu-NPs. Antibacterial copper(II)-chelated PAN membranes were reported by Xu et al. [118] and Xu et al. [121]. PAN membrane was immersed in the PEI solution to form a polyelectrolyte layer, and, finally, the CuAc_2_ solution promoted copper immobilization due to the PEI-Cu(II) complex in solution can be cross-linked on membrane surface [118]. Morphology results reported that the micro-porous surface of the PAN-PEI-Cu membrane was more compact than the PAN-PEI membrane. The obtained morphology results agree with the UF experiments. PAN membrane is much more permeable (1070 L/m^2^ h MPa) than the PAN-PEI and PAN-PEI-Cu membranes (507 and 594 L/m^2^ h MPa, respectively). Moreover, the authors reported that the immobilization of Cu in PEI caused a decline in the surface hydrophilicity (contact angle 52.7° > 70.7°), but the PAN-PEI-Cu membrane showed higher water permeability despite its lower surface hydrophilicity. PA-PEI-Cu membrane presented a good rejection (91%) towards HA (at 5 mg/L); the antibacterial efficiency against *E. coli* was of 71.5% [118]. Xu et al. [121] reported that using cross-linked PAN/PEI-Cu(II) membrane is able to modulate the release of Cu^2+^, that is around below the threshold of 2 mg/L (WHO guidelines for drinking water). Thanks to the release of Cu^2+^, the antibacterial efficiency was about 95%, while the biofilms formation was prevented for up to six months of testing.

**Figure 11:**
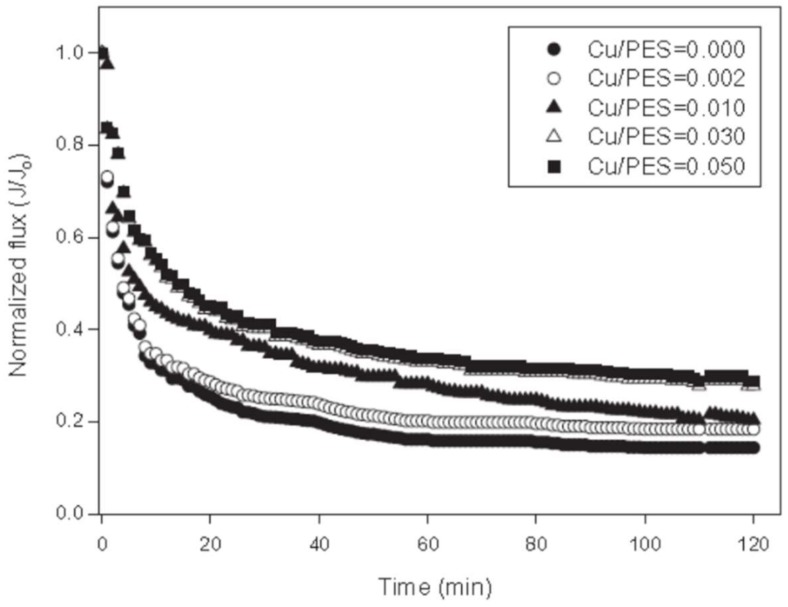
The bio-fouling properties of Cu containing PES composite membranes, adapted from Akar et al. [119].

An interesting study on the effect of polymer concentration (14–18 wt %), time of solvent evaporation (0–90 s) and Cu-NPs concentration (0.002–0.05 wt %), for the preparation Cu-NPs-PES membranes, was reported by Akar et al. [119]. As expected, the water permeability decreased, from 606 to 231 L/m^2^ h bar, when polymer concentration increases from 14 to 18 wt %. To increase the permeability, the authors proposed that the addition of NPs could cause changes in membrane pore size structures and surface electric properties, attributed to the better hydrophilicity and higher density of electrostatic charges on the surface of the Cu-NPs. The antifouling property was evaluated using activated sludge (as a foulant solution), and the nanocomposite membrane loaded with 0.05 wt % showed than better antifouling performance compared to the neat one. As Figure 11 shows, the Relative Flux Reduction (RFR) of neat PES membrane was 93.8% while the RFR of the nanocomposite membranes decreased with increasing the NPs concentration up to 76.2%.

**Figure 12:**
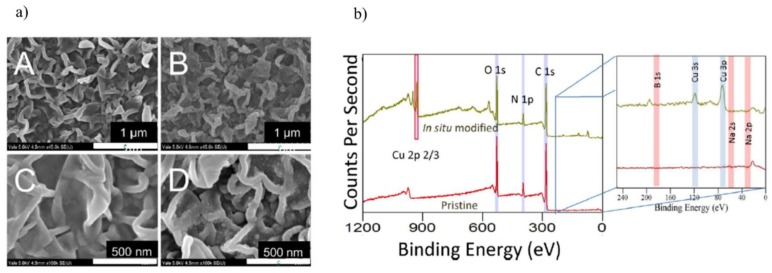
(**a**) SEM images of: (**A**,**C**) pristine; and (**B**,**D**) Cu-NPs TFC-RO membrane; and (**b**) XPS spectra of pristine (red) and modified Cu-NPs TFC membrane (green). The copper peaks are at 932 eV. Adapted from Ben-Sasson et al. [122].

Ben-Sasson et al. [122] reported a novel in situ protocol for loading biocidal Cu-NPs-PA thin film composite membrane for RO. Firstly, the pristine RO membrane was immersed in a CuSO_4_ solution (50 mM) to carry out the impregnation, followed by recoating with NaBH_4_ solution (50 mM) to form Cu-NPs. The Cu-NPs membrane was confirmed by SEM and X-ray photoelectron spectroscopy analysis, as Figure 12 shows, indicating that the Cu-NPs were composed primarily of metallic copper, and to some extent copper oxide.

**Figure 13:**
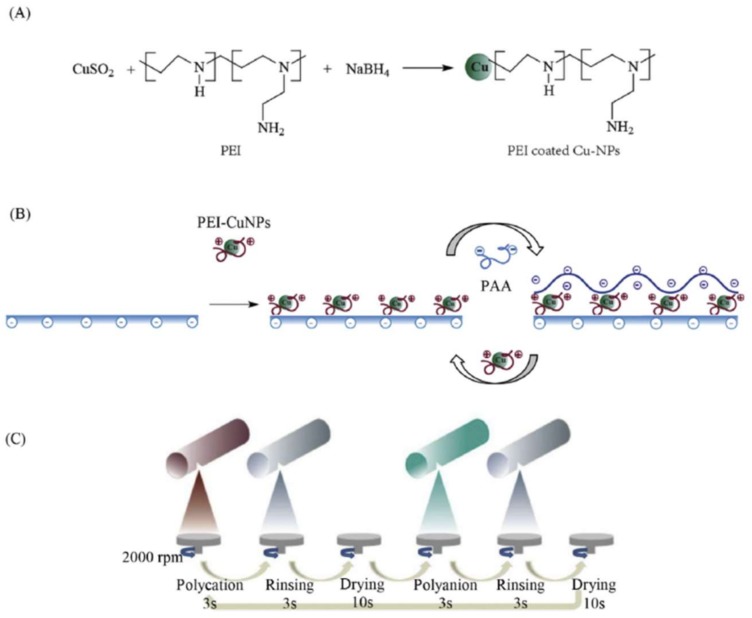
Schemes of: (**A**) preparation of PEI-coated Cu-NPs; (**B**) coating Cu-NPs on the membrane surface via the layer-by-layer self-assembly method; and (**C**) spray- and spin-assisted layer-by-layer (SSLBL) self-assembly process, adapted from Ma et al. [186].

The nanocomposite membranes showed a slight increase in water permeability after the Cu-NPs in situ modification, with values of 2.53 and 2.97 L/m^2^ h bar for the pristine and Cu-NPs membrane, respectively. However, salt rejection (98.31%) decreased slightly for the in situ modified membrane in Cu-NPs membrane, as well as = the number of attached live bacteria reduced compared to the pristine membrane [122]. Similar studies were conducted by Zhang et al. [123] who developed composite membranes with good performance in terms of antibacterial efficiency, water flux and salt rejection. The membranes were obtained by in situ formation of Cu-NPs carboxylated chitosan (CCTS) as a polymer, and glutaraldehyde as a crosslinker agent. The authors suggested that this CuNPs/CCTS modification method provides a simple and low-cost way to enhance both the long-lasting antibacterial and anti-protein-fouling performance. Ma et al. [185] developed a spray- and spin-assisted layer-by-layer method to functionalize thin film composite PA membranes for RO; a simplified scheme of this procedure is described in Figure 13.

Stability experiments demonstrated that the quantity of Cu on the modified membrane surface remained nearly unchanged, indicating a stable binding between the NPs and the membrane surface. During a seven-day test, the release of Cu^2+^ accounted for 29.8% of the total amount of copper on the membrane. Despite water and salt permeability showing a slight decrease when increasing the number of the coating layers (from 0 to 10), the inactivation of bacteria increased with increasing Cu-NPs loading on the membrane surface. Moreover, the authors suggested that the quantity of Cu-NPs and the bacterial inactivation properties of the membrane could be regenerated by the same technique after the depletion of Cu-NPs. Finally, the regeneration of Cu-NPs in situ could be a feasible way to enhance the anti-biofouling performance of the RO membrane with lower cost in comparison with others inorganic materials, e.g., Ag-NPs [186].

## 6. Zinc Oxide (ZnO)

Zinc oxide (ZnO) is another multifunctional inorganic nanoparticle that has attracted attention due to its physical and chemical properties including catalytic, antibacterial and bactericide activities. Moreover, ZnO NPs can absorb hydrophilic hydroxyl groups (-OH) [33], while their surface area seems to be higher than other inorganic nanomaterials [187]. The incorporation of this inorganic filler leads to enhance some properties in polymers such as the hydrophilicity, mechanical and chemical properties [185,188]. For example, the addition of ZnO generates higher hydrophilicity on PES nanofiltration membranes, resulting in higher permeability of the ZnO-filled nanocomposite membranes. Additionally, the use of the filler enhanced the fouling resistance during the filtration of solutions containing humic acid (HA), a typical foulant in natural waters [38]. An enhancement of fouling resistance in ZnO-PES composites membranes, coupled with the improvement on hydrophilicity, thermal properties and water permeability, was observed by Shen et al. [185]. PSF membranes were also filled using this inorganic material demonstrating an antifouling effect on membrane surface as well as inside the pores of the composite membranes [187]. Liang et al. [54] prepared PVDF nanocomposite membranes filled with ZnO NPs. The prepared membranes showed a 100% initial water permeability recovery after membrane cleaning, which means that the irreversible fouling feature, generally encountered in membrane processes, has been avoided. Nanocomposite membranes based on polyvinylpyrrolidone (PVP) and ZnO also displayed antifouling properties during the removal of methylene blue and HA in water purification [69]. ZnO possess the ability to be mixed with other fillers, such as graphene oxide, to form hybrid GO-PSF [72], these ZnO-GO composite membranes are more hydrophilic compared to other GO-PSF membranes, exhibiting excellent antifouling and antibacterial properties during the filtration of HA solutions.

Definitely, the enhancement on fouling resistances adding ZnO on polymeric membranes can be a way to extend the shelf life of the membranes [189]. On the other hand, the addition of ZnO NPs into membranes for the treatment of wastewaters also tends to show high removal efficiencies of heavy metals [70]. Such absorbent capacity is totally attributed to ZnO materials due to their electropositive nature [190,191].

Finally, satisfactory results have been demonstrated through the incorporation of ZnO into polymeric membranes to fabricate nanocomposites membranes with better surface hydrophilicity translated to better performance in terms of permeability [192].

## 7. Graphene Oxide (GO)

Graphene oxide (GO) is a carbon nanomaterial that is obtained in oxidized form of graphene. It exhibits a hydrophilic nature, unlike graphene, which is hydrophobic [189]. This nanomaterial can improve thermal and mechanical properties of polymeric membranes [193]. Typically, GO presents functional groups which provide a variety of surface-modification reactions, i.e., carrying various hydrophilic functional groups (-NH_2_, -OH, and -SO_3_H) [194,195], that can be useful to produce functionalized graphene oxide- and graphene-based materials [196]. GO has recently attracted attention in nanocomposite membranes preparation for application in water treatment including water desalination, and removal of toxic ions and organic molecules in polluted water [197], and it is actually considered as one of the potential nanomaterials applied for the removal of pharmaceutical traces from water and wastewater [198]. Similar to other nanofillers previously mentioned, the incorporation of GO can also carry out the change of the hydrophobic profile to hydrophilic in polymeric membranes tending to improve the permeability performance [84]. Chang et al. [81] investigated the synergistic effects of GO and PVP on PVDF ultrafiltration membrane performance. The results of this investigation showed that the membrane hydrophilicity and the antifouling performances were improved by the addition of GO and PVP. The authors reported that this improvement is due to the formation of hydrogen bonds between PVP and GO.

Yang et al. [90] improved the water flux and antifouling property of poly(m-phenylene isophthalamide) (PMIA) nanofiltration membrane through adding GO; this composite membrane also showed at least 90% dye retention. The use of GO with other fillers, such as oxidized-MWCNT, tends to offer also a synergistic effect on antifouling properties in PVDF polymeric membranes [199]. Zhang et al. [200] also evaluated MWCNTs-interlinked GO composite membranes supported on other polymer such as PAN; these composite membranes were tested for the treatment of strontium-containing wastewater, displaying higher flux compared to other NF membranes. In addition, the membranes could reject around 93% of EDTA-chelated Sr^2+^ in an alkaline solution. Finally, the authors concluded that such membranes are suitable to separate Na^+^/Sr^2+^ mixtures [200]. GO-TiO_2_ composite was assembled on the surface of CA membrane; this membrane displayed high permeate flux and no fouling (with HA solutions) as well [201]. In fact, the use of GO-TiO_2_ composite films are also useful for water purification thanks to their capability in removing dye molecules (methyl orange and rhodamine B) from water [202]. The blending of copper oxide (Cu_x_O) and GO nanofillers based on PVDF was proposed as a potential composite material for water treatment [203]. The filler incorporation enhanced the pristine polymer in several ways: (i) the antifouling property in terms of irreversible fouling based on the enhanced hydrophilic profile of the composite membrane; (ii) antibacterial activity; and (iii) high effective permeability.

In a possible application of desalination, Ganesh et al. [88] evaluated the salt rejection of GO-filled PSF membranes, the NF composite membranes achieved to remove up to 72% of Na_2_SO_4_ from the feed solution. Moreover, the water flux was improved in comparison with the pristine polymeric membrane.A GO-filled poly(amide-imide)-polyethyleneimine (PAI-PEI) membrane also demonstrated high salt rejections (NaCl: 60%; CaCl_2_: >95%), pointing out its potential utility for water softening applications [89]. Polypiperazine-amide (PPA) composite NF membrane with GO displayed high rejections for different salts in the order: Na_2_SO_4_ (98.2%) > MgSO_4_ (96.5%) > NaCl (56.8%) > MgCl_2_ (50.5%) [93].

On the other hand, the grafting of diallyldimethylammonium chloride (PDADMAC) in GO has achieved a potential functionalization of the nanomaterial due to the NPs enhanced the performance of PSF membranes leading the removal of some specific compounds such as heavy metals, salts (NaCl, Na_2_SO_4_, K_2_SO_4_, and MgCl_2_) [204]. For example, a 7 wt % GO-PDADMAC/20% PSF membrane showed high rejections of 86% and 88% towards Cu^2+^ and Cd^2+^, respectively. Likewise, the modification of GO has demonstrated an enhancement on hydrophilicity and fouling resistance, i.e., isocyanate-treated GO was added to PSF membrane [80]. Moreover, an enhanced smoothness of the membrane surface was observed which prevents the protein adsorption meaning better fouling resistance. The negative surface charge displayed by the NPs plays an important role because they can repel the foulants having negative charge [205]. Organosilane-GO was incorporated in PVDF UF membranes [206], the PVDF/functionalized-GO membranes exhibited higher hydrophilicity, water flux, and protein rejection than pristine PVDF membranes and GO-filled-PVDF membranes. The composite membranes also displayed better antifouling properties based on their higher hydrophilicity [206]. Recently, Zhang et al. [207] cross-linked a composite GO with isophorone diisocyanate (IPDI), and then coated on PVDF MF membrane; the cross-linking procedure improved slightly the removal for dyes (above 96%) and heavy metal ions (Pb^2+^, Cu^2+^, Cd^2+^, and Cr^3+^) (40–70%) in comparison with the pattern GO-PVDF membrane.

A new concept of GO modification for composite membranes was developed by Jiang et al. [208,209]: crumpled-GO nanostructures were applied for water treatment. These structures have inherent physical defects (holes), with high ridges and low valleys, readily forming nanoscale channels for potential fast water transport and permeation. Meanwhile, the attachment of functional NPs, such as TiO_2_ [209], Ag [208], leads the water disinfection. Both studies demonstrated that such crumpled-GO-PES membrane composites offer higher water permeabilities than commercial UF membranes, and excellent rejection rates (>80%) against two model contaminants (serum albumin and methyl orange). The synthesis of MgSi modified-GO-PAN membranes can also reach such removal (>75%) for some organic dyes [210]; in the case of these NF composite membranes, the authors highlighted their possible applications for water purification, which is one of main objectives in water treatment. In addition, the fabrication of GO-silver NPs composite onto CA membrane showed a great potential application not only for water purification but also biofouling control in membrane filtration [211].

In a different scope, aiming at water purification, the use of GO-based-composite membranes for water desalination through RO has been investigated [212]. For example, Yin et al. [213] enhanced polyamide (PA) by producing GO thin-film nanocomposite (TFN) membrane for water purification, high rejections about of 95% and 98% were obtained for NaCl and Na_2_SO_4_, respectively. Similarly, an almost complete NaCl removal (>99%) was reported by Chae et al. [94] in the preparation of TFN based on GO-PA membranes; in addition, the incorporation GO improved also the water permeability and anti-biofouling property [94]. Similar rejections (rejection > 97%) were reported by Ali et al. [96] and He et al. [95] during the removal of NaCl and Na_2_SO_4_, respectively, using TFC-based PSF membranes embedded with GO. Furthermore, Ali et al. [96] reported that their membranes demonstrated good stability in acid and alkaline environments.

## 8. 2D Materials

Other materials, such as “2D” materials, have been used for the synthesis of nanocomposites as new concept for the design of membranes for water treatment [98]. Some materials classified in this category are graphene, clay, di-vanadium penta-oxide (V_2_O_5_), zeolite MCM-22, and MCM-41 silica, just to mention a few. These materials have attracted the attention based on their large and chemically active surface area, which contained in composite membranes, can significantly enhance the transport properties based on their unique shape, size, and structure [214]. Moreover, the advantageous transport properties of 2D materials could encourage new opportunities to the synthesis of nanoporous desalination membranes [214,215]. In water treatment, graphene is one of the primary 2D materials that shows a promising future based on its features, such as honeycomb lattice structure, which leads the non-permeability to many molecular components due to the position of electrons within aromatic rings. The electrons form a faultless barrier to the passage of molecules and at the same time they offer an ease permeation of water molecules. Additionally, the use of graphene has been exploited mainly for two types of water treatment: (i) water desalination [216]; and (ii) water purification [217]. In the case of water desalination, it demonstrated a high feasibility though RO technologies; the nanomaterial has shown high salt rejections ranging from 33% to 100% [216]. Meanwhile, the application of graphene for water purification permits efficient removal of organic dyes; for example, graphene-coated on PVDF shows high retentions (>99%). On the other hand, the graphene-based membranes were also evaluated for different salt rejections, their performances were around 20–60%, following the order: Na_2_SO_4_ > NaCl > MgSO_4_ > MgCl_2_ [99].

The performance of graphene has been recently analyzed for wastewater treatment approaches as well. For example, Yang et al. [218] developed a graphene-containing composite membrane for the removal of phthalates such as di(2-ethylhexyl) phthalate and di-n-butyl phthalate and pharmaceuticals (e.g., caffeine, sulfamethoxazole, and cephalexin) from aqueous solutions. The composite membrane showed high removal efficiencies about of 99% for di-n-butyl phthalate and di (2-ethylhexyl) phthalate, and 32–97% for cephalexin, sulfamethoxazole and caffeine. Crock et al. [98] prepared polymer composites with graphene-based hierarchical fillers; basically, graphite nano-platelets were decorated by AuNPs, and then, incorporated to PSF. These composite membranes demonstrated a significant improvement on rejection (from 28% to 69%) for compounds with a molecular weight around 12 kDa (i.e., dextran), while the water permeability increase 22-fold compared to pristine PSF membrane. The results show that the potentiality of these membranes based on graphene for water treatment is highly relevant.

Another 2D material, which has been tested in the preparation of composite membranes, is di-vanadium penta-oxide (V_2_O_5_). Gao et al. [217] prepared a novel hierarchical TiO_2_/V_2_O_5_ multifunctional membrane for potential application in water purification, the novel membranes exhibited a high efficiency removal on TOC removal (up to 90%) in the water filtration, high water flux, and reduce membrane fouling (with HA solutions). Additionally, the membrane was used in a photodegradation process for organic water pollutants, making the membranes even more capable for water purification.

## 9. Some Other Novel Nano-Sized Materials

Currently, various inorganic fillers in the range of nano-scale, e.g. MCM-41 silica, SiO_2_ [219], zeolite MCM-22 [220], clays [47], alumina (Al_2_O_3_) and Fe_3_O_4_, have started to be incorporated into polymeric membranes aiming at water purification and desalination, or wastewater treatment through using NF and RO technologies. For example, zeolites are crystalline alumina-silicate materials with a three-dimensional framework structure. Commonly, zeolite membranes have been used for desalination, gas and liquid separations and membrane reactors [221,222,223,224,225]. Several studies demonstrated that zeolite membranes possess unique properties for different applications, displaying high permeate fluxes and selectivity, while excellent thermal and mechanical stabilities were also obtained [226]. In addition, the incorporation of zeolite NPs into nanocomposite membranes is advantageous due to the enhanced hydrophilicity and molecular-sieving; recently, a layer-by-layer (LbL) assembly approach to incorporate zeolite NPs was presented by Kang et al. [227]. The authors used two different polyelectrolyte layers, negative (PAA) and positive (PEI), and negatively charged Linde type A (LTA) zeolite NPs to form a PEI-LTA-PAA tri-layer using PAN as support. Before the LbL assembly process, the PAN support was coated with a layer of polydopamine (PDA), and membranes were prepared by first dipping the support in PEI, then LTA, and finally in PAA solutions. This cycle can be repeated to create multiple (up to six) tri-layers. Membrane performance was tested in an FO/PRO membrane system, using 1 M MgCl_2_ or 0.5 M trisodium citrate (TSC) as the draw solution and DI water as feed solution. Polyelectrolyte bilayer membrane without zeolite was used as white to highlight the effects of zeolite on membrane performance. As reported, incorporation of zeolite NPs s enhanced membrane water flux of 4.8 μm/s, compared to 2.2 μm/s in bilayer membrane, and the solute rejection was improved as well. The authors suggested that the increase in water flux without a decrease in selectivity indicates that the incorporation of porous zeolite NPs extremely hydrophilic, into the polyelectrolyte layers, creates paths for fast water transport. Moreover, one important advantage of using LbL is the possibility to tailor the membrane surface properties by altering the last deposition layer, and consequently the charge, to achieve better water flux, selectivity, and fouling resistance.

On the other hand, several authors reported the incorporation of zeolite NPs via interfacial polymerization (IP) [228,229,230,231]. An interesting study on the performance and stability of two types of zeolite NPs on the thin film composite RO membranes, was described by Huang et al. [232]. The authors compared the chemical stability in terms of acid and multivalent cation tolerance, of NaA and silicalite-1 zeolite on desalination applications. The obtained data showed that silicalite-1 membrane has better capacity to enhance membrane permeability than NaA. The authors reported that silicate-1 (an alumina-free zeolite), thanks to its properties, is non-susceptible to acid and multivalent cations; on the contrary, NaA will undergo a de-alumination reaction due the existence of alkaline Al in the crystal. The membrane prepared with 0.05 wt % of silicate-1 gave the best results in the RO permeability tests (2000 ppm NaCl), with a value of 66.6 L/m^2^ h bar compared to 29 L/m^2^ h bar for NaA membrane prepared at the same concentration. Furthermore, silicate-1 membrane showed a good NaCl rejection (96.4%) at a certain zeolite concentration (between 0.012–0.025 wt %), and even more in the multivalent cation (CaCl_2_) tolerance test, the separation performance remained unchanged as there was no ion-change activity with the silicalite-1 zeolites. In fact, during the tolerance test, NaA membrane showed a decrease in rejection and enhanced permeability. The authors suggested that this trend can be attributed to an ion exchange process with Na^+^ and Ca^2+^ ions that increase NaA membrane pore size. More recently, Hang et al. [233] investigated the effects of IP reaction time and zeolite loading on membrane separation performance, demonstrated as a longer IP reaction time (20 s) increase the membrane thickness and cross-linking degree, leading to a lower water flux and a higher salt rejection of 1.53 m^3^/m^2^ day and 98.9% respectively. Moreover, at 0.15 wt % zeolite, the composite membrane showed the best separation performance in terms of both water flux (1.78 m^3^/m^2^ day) and salt rejection (98.8%). Finally, Dong et al. [231] proposed a novel approach in order to prepare thin film nanocomposite NF membranes, using PS support in situ embedded with zeolite NPs followed by IP to form the PA layer. These new membranes presented a coverage ratio of NPs (up to 49%), higher water permeability, and good salt rejection of 93.4%.

Silicon dioxide or silica (SiO_2_) is another inorganic metal oxide material, which has been used as filler based on its advantages such as convenient operation, lower reactivity and good chemical properties. For instance, Ahmad et al. [234] prepared functionalized-PS membranes by blending with different SiO_2_ concentration (0–5 g). The hydrophilic property of the SiO_2_ allowed to increase the permeate flux up to 17.32 L/m^2^ h in contrast with the unmodified membrane (1.08 L/m^2^ h). The results obtained from the antifouling performance, which was evaluated in terms of oil-in-water emulsion separation, indicated lower oil droplets deposition due to the enhancement of hydrophilicity by SiO_2_ NPs. Similarly, Huang et al. [235] also observed this trend when mesoporous silica (MS) was used for PES nanocomposite membranes. The MS increased the pore size in the sub-layer and improved the interconnectivity of pores between the sub and bottom layer. Particularly, the MS particles enhanced the hydrophilicity of the membrane displaying a flux of 180.2 L/m^2^ h and BSA rejection around 96.1% at 2 wt % filler loading.

On the other hand, the effect of SiO_2_ NPs on morphology and performance of thin film nanocomposite membranes for FO application have been investigated by Niksefat et al. [236]. In principle, PA nanocomposite membranes containing different concentration of silica nanoparticles, from 0.01 to 0.1 wt %, were synthesized via in situ IP. The performance of composite membranes was compared to thin film membranes without NPs. The morphological analysis showed that the SiO_2_ NPs were attached to the FO membrane surface, showing that membranes have ascendant and broadened ridge valley structure with rougher surface than the unmodified membranes; furthermore, the surface roughness increased by adding SiO_2_ NPs. In the case of FO water and salt experiments, it was confirmed that flux increase with increasing silica loading, e.g., water permeability up to 12.36 (×10^−12^ m/spa) with a 78% of NaCl rejection for composite membranes containing 0.1 wt % SiO_2_. Kebria et al. [237] also prepared SiO_2_-PEI thin film composite NF membranes, for dye removal from aqueous and organic solution. Basically, PEI and two different concentrations of triphthaloyldechloride (TPC) (0.1 and 0.5 wt %) were used for the interfacial polymerization reaction in the presence of different contents (0.03, 0.05, and 0.1 wt %) of SiO_2_. For aqueous solution, higher flux of 13.3 L/m^2^ h and rejection of 100% of crystal violet as cationic dye were obtained using 0.1 wt % SiO_2_ NPs and 0.1 wt % TPC. Recently, Zha et al. [238] studied the desalination of water from oilfield wastewater; in particular, SiO_2_/PES hollow fibers via IP were prepared and tested by using feed solution containing various salts such as NaCl, Na_2_SO_4_, MgCl_2_ and MgSO_4_. The results showed that the permeate water flux and ions rejection of the hollow fibers increased linearly with the pressure from 50 to 90 psi, but at 100 psi water flux still increases and rejection slightly declined. In the case of water flux, it decreased in the order of Na_2_SO_4_ > NaCl > MgCl_2_ = MgSO_4_ for feed solutions at the same concentration, and rejection values decreased in the order of Na_2_SO_4_ > MgSO_4_ > MgCl_2_ > NaCl. This trend indicated that salt rejection was not only affected by the total ion concentration, but also the ions’ sizes. Finally, the authors reported that, when operating pressure was 80 psi, after 56 h water flux reaches the steady state and the decrease of water flux was mainly caused by membrane fouling during the NF process.

Aluminum oxide (Al_2_O_3_) NPs are well known for increase the hydrophilicity and suppress the fouling in polymeric membranes [239,240,241,242,243]. Indeed, several preparation methods has been reported in literature, such as IP [239], surface deposition and structure entrapment [240] and in situ polymerization via sol-gel processes [241]. Actually, the performance of Al_2_O_3_-filled nanocomposite membranes was compared to other metal oxide particles e.g., TiO_2_ and ZrO_2_ [42]. The effect of these different fillers in terms of type, size, and distribution on the final membranes was investigated. The obtained data showed that Al_2_O_3_-PES membrane exhibited the highest particle number near the membrane surface that because Al_2_O_3_ is less dense than other selected metal oxides (ZrO_2_ > TiO_2_ > Al_2_O_3_). The authors reported that during phase separation process, the solvent driven by the diffusive transfer drags the added particles and during the casting, some particles could precipitate to the support. Furthermore, as a consequence of pore formation and hydrophilicity enhancement, Al_2_O_3_-PES membrane exhibited the highest water flux value of 208.9 L/m^2^ h. Concerning to the fouling testing, higher flux was still observed for Al_2_O_3_-PES membrane. The authors explained this trend could be attributed to the fact that Al_2_O_3_ has more hydrophilic centers (metal oxide particles) in the vicinity of membrane surface, which reduces the possible adsorption of foulants (e.g., HA). Similar results were observed during tests with BSA, in fact, the high density of Al_2_O_3_ NPs on the membrane surface prevents the deposition/adsorption of BSA molecules on the membrane inhibiting the formation of a fouling layer [42].

Iron oxide type II and III (Fe_2_O_3_ and Fe_3_O_4_, respectively) presents many outstanding features including magnetic property, antibacterial and antifungal activities, capacity to absorb hydrophilic hydroxyl groups to become hydrophilic. Zinadini et al. [92] reported the effect of Fe_3_O_4_ NPs on PES membranes for dye removal. In this study, O-carboxymethyl chitosan bound Fe_3_O_4_ magnetic nanoparticles (CC-Fe_3_O_4_ NPs) were synthesized via co-precipitating method to prepare nanocomposite PES-CC-Fe_3_O_4_ membranes with different filler concentration, from 0 to 1 wt %. Practically, thin layer was obtained at 0.1 wt % of CC-Fe_3_O_4_, but more than 0.5 wt % of the CC-Fe_3_O_4_ NPs tended to form a denser skin-layers with increased in porosity. However, the best water flux (32 kg/m^2^ h) with 99% dye rejection was obtained at such filler loading. On the other hand, the first detailed work related to the use of Fe_2_O_3_ particles in the synthesis of UF membranes was reported by Demirel et al. [244]. The influence of Fe_2_O_3_ NPs dispersed in PVC polymer on the membrane performance in terms of rejection and antifouling properties has been investigated. Fe_2_O_3_-PVC membranes with varying amounts of NPs (0–2 wt %) were prepared via phase inversion techniques. Specifically, the best water flux increased from 522 to 782 L/m^2^ h at 1 wt % in comparison to the unfilled one, displaying additionally a SA rejection of 91.6% with flux recovery around 91.5%.

## 10. Concluding Outlook

Generally, the addition of different classes of NPs (ZnO, Ag, Cu, GO, TiO_2_, graphene, Al_2_O_3_, Fe_3_O_4_, zeolite, clay, and SiO_2_) into polymeric membranes tends to enhance the hydrophilicity of polymeric membranes, reducing fouling phenomenon in water treatment. Moreover, some other properties (mechanical, thermal, and chemical) of the polymers are enhanced as well. In the case of GO, it tends to form nanocomposite membranes which present a higher hydrophilicity and fouling resistances, but its chemical modification through several mechanisms leads to obtain promising results for the removal of specific compounds, i.e., heavy metals. TiO_2_ particles have been exploited enough but their uses are additionally addressed as composites with other fillers, e.g., GO, CNTs, SiO_2_ and Ag. On the other hand, CNTs have been functionalized which can enhance the chemical properties aiming enhancements on the hydrophilicity while improving the mechanical properties. These approaches provide an outlook of the real potentialities of these smart membranes, for example, in water purification. In particular, the versatility of composite membranes for an efficient water treatment, such as wastewater processing, can be enhanced through their coupling with other technologies; for example, photocatalytic process [86,245,246], electrocoagulation, electrofiltration [218], or membrane bioreactor [148,149].

In fact, nanomaterials possess unique size-dependent properties related to their high specific surface area; this feature contributes to the development of novel high-tech materials for more efficient water and wastewater treatment processes [247]. However, the extensive use of nanomaterials depends on the potential risk involved and on the cost effectiveness. The World Health Organization reported in their guidelines the limit and the risk related to the use of NPs, as reported in this work [164]. The approach for minimizing the release of NPs during membrane process is to control the effective adhesion on the polymeric matrix and valuated the stability during time. Therefore, one of the challenges is to develop easy and low-cost methods to immobilize NPs on membranes without reducing their performance [248]. Many studies focus on the release of NPs, which is a major technical hurdle for risk assessment, and the detection techniques, which are few, usually sophisticated, expensive and with many limitations] [249]. However, what today represents a problem is only a small obstacle to be overcome, considering that academics, governments and companies are working in this direction. Finally, development of novel nanocomposite membranes has proven to be successfully applied in water treatment, thanks to their antifouling and antibacterial properties, with the objective of enhancing the membrane lifetime and as well as separation performance.

## Figures and Tables

**Table 1:** Application of different nanoparticles into polymeric membranes for water treatment.

Nanoparticle	Membrane Process	Application	Polymer	Filler Concentration:	Reference:
ZnO	MF	Treatment of synthetic wastewater	PVDF	6.7–26–7 wt %	[54]
		Removal of copper ions		1–5 wt %	[55]
		Removal of COD from wastewater		0–1 wt %	[56]
		Removal of HA	PES	3.6 wt %	[57]
	UF	Removal of HA	PSF	0.1 wt %	[58]
		Removal of salt	PA	0.003–0.009 g	[59]
		Evaluation of antifouling properties in composite membranes for water treatment. Mixture model: BSA	PVDF	1 g	[60]
		Evaluation of antifouling properties in composite membranes for water treatment. Mixture model: BSA	PES	0.5–2 wt %	[61]
		Removal of micelle from aqueous solutions		0–10 wt %	[62]
		Removal of pollutants Sodium alginate, BSA and humic acid (HA)		0.25–0.75 wt %	[63]
		Evaluation of antifouling properties in composite membranes for water treatment. Mixture model: BSA		0.4 g	[64]
		Evaluation of antifouling properties in composite membranes for water treatment. Mixture model: BSA	PES-PVA	0.04–1.3 g	[65]
		Treatment of wastewaters	PSF	0.1–1 wt %	[66]
		Bacterial removal from aqueous solutions		0.7 mg	[67]
		Evaluation of antifouling properties in composite membranes for water treatment. Mixture model: BSA	PVC	3 wt %	[68]
	NF	Removal of HA	PES	0.035–4 wt %	[38]
		Water purification (removal of HA)	PVP	100 mg	[69]
		Removal of salt and metal ions (Zn^2+^, Cd^2+^, Pb^2+^, Mn^2+^, Ni^2+^, Fe^2+^, Al^3+^, Sb^3+^, Sr^3+^)	CA	0.02–0.05 g	[70]
		Separation of Rhodamine B	CTA	0.6 g	[71]
		Removal of HA	PSF	2 wt %	[72]
		Removal of inorganic salts and HA	PVDF	0–0.2 wt %	[73]
		Removal of HA		1 wt %	[74]
		Removal of salts (model MgSO_4_)	Poly(piperazine amide)	1.5 wt %	[75]
	FO	Desalination and water treatment	PVDF	0–8 wt %	[76]
	RO	Removal of salt, bivalent ions (Ca^2+^, SO_4_^2−^ and Mg^2+^), monovalent ions (Cl^−^ and Na^+^), and bacterial retention	PA	0.005–0.4 wt %	[77]
GO	MF	Treatment of effluents with high dyes content	PSF	0.75–2.5 wt %	[78]
		Filtration of wastewaters	PVDF	3 wt %	[79]
	UF	Evaluation of antifouling properties in composite membranes for water treatment Mixture model: BSA	PSF	0.025–0.15 wt %	[80]
		Evaluation of antifouling properties in composite membranes for water treatment Mixture model: BSA	PVP-PVDF	0–0.50 wt %	[81]
		Evaluation of antifouling properties in composite membranes for water treatment Mixture model: BSA	PVDF	2.5 g/mL	[82]
		Natural organic matter removal		0.1–1 wt %	[41]
		Evaluation of antifouling properties in composite membranes for water treatment Mixture model: BSA		0–2 wt %	[83]
		Natural organic matter removal	PA	0.004–0.012 wt %	[84]
		Wastewater treatment	PSF	0.02–0.39 wt %	[85]
		Degradation of organic pollutants in salty water	Cellulose ester	2 g/L	[86]
		Treatment of distillery effluent	PES	0.5–1 wt %	[87]
	NF	Na_2_SO_4_ rejection from water streams	PSF	2000 ppm	[88]
		Water softening production	PAI-PEI	5 mg/mL	[89]
		Treatment of effluents with high dyes content	PMIA	0.05–0.5 wt %	[90]
		Treatment of solutions with high dyes content	PAN	0.25–1 g/L	[91]
		Evaluation of dye removal capacity for water treatment	PES	0.1–1 wt %	[92]
		Water purification	PPA	100–400 mg/L	[93]
	RO	Desalination: Salt removal (NaCl)	PA	5–76 ppm	[94]
		Desalination: Salt removal (NaCl, CaCl_2_ and Na_2_SO_4_)	PSF	0.005–0.3 wt %	[95]
		Desalination: Salt removal (NaCl)		100–300 ppm	[96]
	FO	Possible prospect for desalination of sea water	PA	1.5 wt %	[97]
Graphene	UF	Wastewater treatment	PSF	0.1–2 wt %	[98]
	NF	Water purification	PVDF	0.864 μg/mL	[99]
AgNO_3_	UF	Reduction of the microbial load of raw milk during the concentration process by the UF process	PES	2–4–6 wt %	[100]
		Evaluation of antifouling properties in composite membranes for water treatment. Mixture model: BSA	PSF	0.5 wt %	[101]
AgNPs	MF/UF	Evaluation of antifouling properties in composite membranes for water treatment. Mixture model: BSA		0–0.05–0.1–2.5–5–10 wt %	[102]
	UF	Water purification	PES	0–0.32–0.64 wt %	[103]
		Evaluation of antifouling and antibacterial properties in composite membranes for water treatment. Model bacteria: *E. coli*	PES, PSF, CA	0.03–0.06–0.09 wt %	[104]
		Evaluation of antifouling and antibacterial properties in composite membranes for water treatment. Model bacteria: *E. coli*. Mixture model: BSA and dextran solution	PSF	0.25–0.5–1.0 wt %	[105]
		Evaluation of antifouling and antibacterial properties in composite membranes for water treatment. Model bacteria: *P. putida*. Mixture model: BSA		3.6 gr	[106]
		Evaluation of antifouling properties in composite membranes for water treatment Mixture model: polyethylene glycol (PEG) and Dextran solutions	CA	0–0.1–0.4 wt %	[64]
	NF	Evaluation of antibacterial properties in composite membranes for water treatment Model bacteria: *E. coli*, *S. aureus*		0.5–1–2 wt %	[107]
Ag-NO_3_		Evaluation of antibacterial properties and removal of salt (Na_2_SO_4_). Model bacteria: *E. coli*	PA-PVA	10 mL	[108]
	RO	Evaluation of antibacterial properties and removal of salt (NaCl). Model bacteria: *E. coli*, *P. aeruginosa*, *S. aureus*	PA	10 mL	[109]
		Evaluation of antibacterial properties and removal of salt (NaCl). Model bacteria: *E. coli*, *Bacillus subtilis*	PA/PSF/PET	4 g/L	[110]
		Evaluation of antibacterial properties Model bacteria: *E. coli*, *Bacillus subtilis*	CA	-	[111]
	DCMD	Deposition of silver nanoparticles layers to optimize surface roughness and enhance membrane hydrophobicity. Desalination of seawater. Model water: NaCl 3.5 wt %	PVDF	1 wt %	[112]
	PRO/RO	Evaluation of antifouling and antibacterial properties in composite membranes for water treatment. Model bacteria: *E. coli.* Mixture model: BSA	PES	40 g/L	[113]
Ag-NPs	PRO	Evaluation of antifouling and antibacterial properties in composite membranes for water treatment. Model bacteria: *E. coli*, *Bacillus subtilis* Mixture model: *C. testosteroni*	PAN	0.01–0.02–0.05–0.10 wt %	[114]
bio-Ag_0_	UF	Evaluation of antifouling and antibacterial properties in composite membranes for water treatment. Model bacteria: *E. coli*, *P. aeruginosa*	PES	0.1–0.3–0.5–1 wt %	[115]
	NF	Evaluation of antibacterial properties and removal of salt (Na_2_SO_4_). Model bacteria: *E. coli*, *P. aeruginosa*	PA	0.1 mM 40 mL	[116]
		Evaluation of antibacterial properties and removal of salt (Na_2_SO_4_). Model bacteria: *P. aeruginosa*	PSF	0.005–0.025–0.05 wt %	[117]
Cu-NPs	UF	Evaluation of antifouling and antibacterial properties in composite membranes for water treatment. Model bacteria: *P. putida.* Mixture model: BSA		3.6 g	[106]
CuAc2		Evaluation of antifouling and antibacterial properties in composite membranes for water treatment. Model bacteria: *E. coli.* Mixture model: HA	PAN/PEI	1000 mg/L	[118]
Cu-NPs		Treatment of wastewaters (sludge filtration) and evaluation of antifouling properties in composite membranes for water treatment. Mixture model: BSA	PES	0.002–0.01–0.03–0.05 wt %	[119]
Ag-NPs Cu-NPs		Evaluation of antifouling and antibacterial properties in composite membranes for water treatment. Model bacteria: *E. coli*. Mixture model: PEO	PSF	3.2 g	[120]
CuSO_4_	NF	Seawater softening, removal of salt (SO_4_^2+^, Mg^2+^, Na^+^, Cl^−^). Evaluation of antibacterial properties in composite membranes for water treatment. Model bacteria: E. coli	PAN/PEI	0–0.4 g	[121]
	RO	Evaluation of antibacterial properties and removal of salt (NaCl). Model bacteria: *E. coli*	PA	50 mM	[122]
CuCl_2_		Evaluation of antifouling and antibacterial properties in composite membranes for water treatment. Model bacteria: *E. coli*. Mixture model: BSA		30 mL	[123]
Cu-NPs		Evaluation of antibacterial properties in composite membranes for water treatment and removal of salt (NaCl). Model bacteria: *E. coli*, *P. aeruginosa*, *S. aureus*.		50 mL	[124]
TiO_2_-NPs	MF	Evaluation of antifouling properties using whey solution	PVDF	0.05 wt %	[125]
	UF	Evaluation of antifouling properties in composite membranes for water treatment. Mixture model: HA		0.1 g/L	[126]
		Evaluation of antifouling properties in composite membranes for water treatment. Mixture model: BSA, PEG and MgSO_4_		0.5–1 wt %	[127]
		Treatment of wastewaters		0–0.15–0.3–0.45–1.5–3–6 wt %	[37]
		Evaluation of UV-cleaning properties		0–1.5 wt %	[128]
		Evaluation of UV-cleaning and antifouling properties. Mixture model: BSA		0–7 wt %	[129]
		Evaluation of antifouling properties. Mixture model: BSA and Lys	PP	-	[130]
		Evaluation of antifouling properties and removal of salt (NaCl). Mixture model: BSA and pepsin	PSF	0.1, 0.25 and 0.5 wt %.	[131]
		Water treatment	CA	0–25 wt %	[132]
		Evaluation of UV-cleaning properties and antifouling properties. Mixture model: red dye and BSA.	PA	10–80 ppm	[133]
Titanium tetraisopropoxide (TIP)		Evaluation of antifouling properties. Mixture model: BSA		29.58 mL	[134]
TiO_2_-NPs	FO	Evaluation of removal of salt (NaCl).	PSF	0.01, 0.05, and 0.1 wt/v %	[135]
		Evaluation of removal of salt (NaCl).		0–0.5–0.75–0.99 wt %	[136]
	MF/MBR	Evaluation of antifouling properties. Mixture model: BSA, PEG and MgSO_4_	PVDF	-	[137]
nanoTiO_2_	MBR	Algal membrane bioreactor evaluation		5 wt %	[138]
TiO_2_-NPs	NF	Wastewater treatment application	PES	0.125 g	[139]
CNTs	NF	Evaluation of antifouling and removal of salts (NaCl, Na_2_SO_4_).	PSF	5 wt %	[140]
	NF	Drinking-water purification	Nitrocelullose	3 wt %	[141]
	UF	Water treatment and biofouling control application	PES	0–4 wt %	[40]
	NF	Wastewater treatment application	PES	0.1 wt %	[142]
	NF	Water treatment	PA	5 wt %	[143]
	NF	Metal removal (Cr(VI), Cd(II))	PSF	0.1–1 wt %	[144]
	NF	Water treatment for salt removal (NaCl, Na_2_SO_4_).	PMMA	0.67 wt %	[145]
	NF	Evaluation of antifouling properties in composite membranes for water treatment.	Polyimide 84	0.1–1 wt %	[146]
	UF	Water treatment for UF applications	PSF	0.1–0.5 wt %	[147]
	UF	Wastewater treatment by membrane bioreactor	PSF	0.1–1 wt %	[148]
	MF	Bleach effluent treatment by membrane bioreactor	PSF	0.04 wt %	[149]

## Abbreviations

BSA	Bovine serum albumin
CA	Cellulose acetate
CS	Chitosan
CCTS	Carboxylated chitosan
CTA	Cellulose triacetate
CNT	Carbon nanotubes
CDO	Chemical oxygen demand
FO	Forward osmosis
HA	Humic acid
MMM	mixed matrix membranes
MF	Microfiltration
NP	Nanoparticle
PA	Polyamide
PAA	Poly(acrylic acid)
PAI	Poly(amide-imide)
PAN	Polyacrylonitrile
PEI	Polyethyleneimine
PE	Polyethylene
PEG	Polyethylene glycol
PSF	Polysulfone
PES	Polyethersulfone
PMIA	Poly (m-phenylene isophthalamide)
PMMA	Poly(methyl methacrylate)
PTFE	Polytetrafluoroethylene
PVA	Polyvinyl alcohol
PVDF	Polyvinylidine fluoride
PVP	Polyvinylpyrrolidone
PVC	Polyvinyl chloride
PP	Polypropylene
*E. coli*	*Escherichia coli*
*S. aureus*	*Staphylococcus aureus*
*C. testosterone*	*Comamonas testosteroni*
PRO	Pressure retarded osmosis
PDA	Polydopamine
PVC	Polyvinyl chloride
PPA	Polypiperazine-amide
PDADMAC	Diallyldimethylammonium chloride
NF	Nanofiltration
RO	Reverse osmosis
UF	Ultrafiltration
ZnO	Zinc oxide
GO	Graphene oxide
ED	Electrodialysis
MWCNT	Multi-walled carbon nanotubes
TOC	Total organic carbon
HA	Humic acid
Lys	Lysozyme
DCMD	Direct contact membrane distillation
MBR	Membrane Bioreactor
LbL	Layer-by-layer
LTA	Linde type A zeolite
IP	Interfacial polymerization
TPC	Triphthaloyldechloride
